# Intranasal administration of induced pluripotent stem cell-derived cortical neural stem cell-secretome as a treatment option for Alzheimer’s disease

**DOI:** 10.1186/s40035-023-00384-8

**Published:** 2023-11-09

**Authors:** Hyunkyung Mo, Juryun Kim, Jennifer Yejean Kim, Jang Woon Kim, Heeju Han, Si Hwa Choi, Yeri Alice Rim, Ji Hyeon Ju

**Affiliations:** 1 Present Address: CiSTEM Laboratory, Catholic iPSC Research Center, College of Medicine, The Catholic University of Korea, Seoul, 06591 Republic of Korea; 2 Department of Biomedicine and Health Science, College of Medicine, The Catholic University of Korea, Seoul, 06591 Republic of Korea; 3 YiPSCELL, Inc, Omnibus Park, Banpo-daero 222, Seocho-gu, Seoul, 06591 Republic of Korea; 4 Department of Biology, Georgetown University, 3700 O St NW, Washington, DC 20057 USA; 5 Division of Rheumatology, Department of Internal Medicine, Seoul St. Mary’s Hospital, Institute of Medical Science, College of Medicine, The Catholic University of Korea, Seoul, 06591 Republic of Korea

**Keywords:** Induced pluripotent stem cells, Alzheimer’s disease, Cortical neural stem cells, Secretome, Intranasal administration, Memory disorders, Neuroprotective agents

## Abstract

**Background:**

Alzheimer’s disease (AD) is the most common neurodegenerative disorder in the elderly, resulting in gradual destruction of cognitive abilities. Research on the development of various AD treatments is underway; however, no definitive treatment has been developed yet. Herein, we present induced pluripotent stem cell (iPSC)-derived cortical neural stem cell secretome (CNSC-SE) as a new treatment candidate for AD and explore its efficacy.

**Methods:**

We first assessed the effects of CNSC-SE treatment on neural maturation and electromagnetic signal during cortical nerve cell differentiation. Then to confirm the efficacy in vivo, CNSC-SE was administered to the 5×FAD mouse model through the nasal cavity (5 μg/g, once a week, 4 weeks). The cell-mediated effects on nerve recovery, amyloid beta (Aβ) plaque aggregation, microglial and astrocyte detection in the brain, and neuroinflammatory responses were investigated. Metabolomics analysis of iPSC-derived CNSC-SE revealed that it contained components that could exert neuro-protective effects or amplify cognitive restorative effects.

**Results:**

Human iPSC-derived CNSC-SE increased neuronal proliferation and dendritic structure formation in vitro. Furthermore, CNSC-SE-treated iPSC-derived cortical neurons acquired electrical network activity and action potential bursts. The 5×FAD mice treated with CNSC-SE showed memory restoration and reduced Aβ plaque accumulation.

**Conclusions:**

Our findings suggest that the iPSC-derived CNSC-SE may serve as a potential, non-invasive therapeutic option for AD in reducing amyloid infiltration and restoring memory.

**Supplementary Information:**

The online version contains supplementary material available at 10.1186/s40035-023-00384-8.

## Background

Alzheimer's disease (AD) is a major form of senile dementia, a chronic progressive disease characterized by memory and cognitive impairment leading to death within 10 years of diagnosis [1, 2]. It is estimated that the global prevalence of AD will increase from the current 50 million to 150 million by 2050 with the increase in the size of the elderly population [3]. According to the amyloid cascade hypothesis first advocated by Hardy et al. [4] in the 1990s, amyloid beta (Aβ) is known to be the main cause of AD. However, AD has been reported as a multifactorial disease involving highly phosphorylated tau protein neurofibrillary tangles and the *APOE* gene [5–7]. The AD brain, which has complex neuropathological characteristics, is permanently affected by various inflammatory substances, and the released cytokines create a neurotoxic environment that causes overall brain atrophy, leading to chronic diseases requiring long-term treatment [8, 9]. However, most studies on AD drugs have so far aimed at single pathological targets such as Aβ. In 2021, aducanumab (marketed as “Aduhelm”) was approved as the first disease-modifying agent for treating AD by the Food and Drug Administration; however, it failed to significantly affect cognition in patients with severe form of the disease [10, 11]. When used at a higher dose, a modest impact on the cognitive decline of patients was observed in the early stage of AD or in those with mild cognitive impairment; however, the drug did not reverse prior memory loss associated with AD [10, 12].

Due to the multifactorial pathogenesis of AD, therapeutic strategies targeting multiple targets, rather than single-target therapies targeting molecules such as Aβ or tau, are emerging [13, 14]. In this context, stem cell therapy targeting various pathological mechanisms in AD has emerged as a new approach [15, 16]. The most commonly used stem cell types in recent AD treatment research are brain-derived neural stem cells (NSCs) [17–20] and mesenchymal stem cells (MSCs) [21–23]. Among them, MSCs are commonly used multipotent stem cells with self-renewing and immunomodulatory properties with limited differentiation capacity, mainly observed in mesodermal lineage cells [20, 24]. The anti-amyloid efficacy of MSCs has been proven to repair damaged astrocytes when used as a cell therapy [20, 25, 26]. Despite the reported advantages, various challenges limit the clinical use of MSCs, including the loss of potency and proliferative ability owing to their dedifferentiation and limited lifespan [21, 27]. In addition, studies using existing MSCs have demonstrated an improved anti-amyloid environment, but the limitation of insufficient potency persists [28, 29].

A disease-specific intranasal treatment strategy using stem cells exposed to the AD brain environment has been reported [27, 30]. These studies inspired Alzheimer-specific treatment strategies, which, compared to the existing research directions, rely on the inherent homeostasis of existing stem cells. To select a more AD-specific candidate group, we selected cortical neural stem cells (CNSCs) located in the cerebrum, which perform memory-related and cognitive functions, as more competitive candidates for treatment than MSCs. CNSCs are located in the cortex that holds memory-related and cognitive functions of the brain, and studies suggest that they are more effective than other types of stem cells in treating AD [31].

The aim of this study was to present a potential candidate group of cells as an AD-specific cell-free stem cell-based therapy, by evaluating the paracrine effect of induced pluripotent stem cell (iPSC)-derived cortical neural stem cell secretome (CNSC-SE) that is delivered via intranasal administration [32, 33].

## Methods

### Mice

The Korea Excellence Animal Laboratory Facility of Korea Food and Drug Administration was accredited by the Institutional Animal Care and Use Committee (IACUC) and the Department of Laboratory Animal (DOLA) in the Catholic University of Korea, Songeui Campus in 2017, and acquired AAALAC (Association for Assessment and Accreditation of Laboratory Animal Care) complete international accreditation in 2018. All procedures of animal research were conducted in accordance with the Laboratory Animals Welfare Act, the Guide for the Care and Use of Laboratory Animals, and the Guidelines and Policies for Rodent Experiment provided by the IACUC School of Medicine at the Catholic University of Korea. The animal experiments were approved by the Institutional Animal Care and Use Committee (IACUC) of School of Medicine, The Catholic University of Korea (Approval number: CUMS-2020–0217-08).

The B6.CG-Tg (APPSwFILon on PSEN1*M146L*L286V) 6799Vas/J transgenic (5×FAD) mice which have high amyloid deposition from 2 months of age [34] and C57BL/6 (wild-type, WT) mice were purchased from Jackson Laboratory (Bar Harbor, MN). All mice were maintained under specific pathogen-free conditions. A previous study by Santamaria et al. reported that a single intranasal dose of MSC secretome resulted in sustained memory recovery in AD mice for 7 days [27]. Based on this, we administered iPSC-derived CNSC-SE (5 μg/g) in 12-week-old male mice once a week for 4 weeks (4 times in total) by inserting a 26-G intravenous catheter (Poly Medicure POLYPEN IV Catheter, 26 G/19 mm) along the nasal cavity.

The following four groups were set in this study: (i) WT mice as controls (WT group); (ii) 5×FAD AD mouse model (AD group); (iii) CNSC-SE-treated 5×FAD mice (CNSC-SE-5×FAD group); and (iv) MSC-treated 5×FAD mice (MSC-SE-5×FAD group).

### Barnes maze

The Barnes maze was used to assess spatial learning and memory [35]. The maze consisted of an elevated circular platform (92 cm in diameter), containing 18 equally spaced holes around the circumference, each 5 cm in diameter. Visual cues were placed around the walls (periphery) during the test. For training, a mouse was placed in the center of the maze under a transparent beaker for 1 min, and it was then guided to the target hole, which contained sweet-flavored chips, for 1 min. The mouse was trained for 2 consecutive days followed by testing on day 3. On the test day, the mouse was placed in the center of the maze and observed for 3 min to record the following parameters: (i) distances from the target hole; (ii) the resting time in the target hole; (iii) the first latency to reach the target hole; and (iv) the error rate, i.e., the number of times the mouse entered a zone other than the target zone. After all trials, the maze was completely cleaned with 70% alcohol solution. All experimental tests were recorded by a video camera and analyzed with SMART 3.0 (Panlab, Harvard Apparatus, Barcelona, Spain).

### Cell culture

All hiPSC lines used in this study were generated through our previous research using cord blood cells isolated from a healthy individual [36]. Human iPSCs were seeded on cell culture dishes coated with vitronectin (Gibco, Waltham, MA; #A14700), and cultured in Essential 8 media (Gibco #A1517001) in a 10% CO_2_ environment at 37 °C.

To induce differentiation of iPSCs into cortical neurons, we referred to the protocol of Shi et al. [37]. For neural induction, iPSCs were seeded on vitronectin-coated 60-mm dishes at $4.6\times {10}^{6}$ cells/dish with Essential 8 media and 10 µM rho-associated kinase inhibitor for 24 h. After 24 h of incubation, the culture medium was changed to a neuronal differentiation medium (NDM), which is a 1:1 mixture of N-2 (consisting of Dulbecco’s Modified Eagle Medium, Nutrient Mixture F12 GlutaMAX, 1 × N-2, 5 μg/ml insulin, 1 mM *L*-glutamine, 100 μm nonessential amino acids, 100 μM 2-mercaptoethanol, and 10% penicillin/streptomycin) and B-27 (consisting of Neural basal, 1 × B-27, 200 mM *L*-glutamine and 10% penicillin/streptomycin, with 10 µM SB431542 [Tocris Bioscience, Bristol, UK; #1614]) with 1 µM Dorsomorphin (Tocris Bioscience, #BML-275), and incubated for 10 days. Neuronal progenitor cells were collected by Gentle Cell Dissociation Reagent (STEMCELL Technologies, Vancouver, BC, Canada; #100-0485) for NSC expansion. The cells were seeded on a laminin-coated (Biolamina, Stockholm, Sweden; #LN521-05) 60-mm dish (9 × 10^6^ cells per dish) and cultured with NDM for 6 days, followed by maintenance in NDM supplemented with 20 ng/ml FGF2 for an additional 2 days. After 2 days, the NSCs were gently dissociated with accutase (Innovative Cell Technologies, San Diego, CA; #AT-104) for CNSC expansion and seeded on laminin-coated (Biolamina, #LN111) dishes under the same condition as for NSCs for 6 days. CNSCs, called cortical neurons, were cultured for 5 days and were passaged once more with accutase under the same conditions and maintained for up to 90 days.

Bone marrow-derived human MSCs were purchased from the Catholic Institute of Cell Therapy, South Korea. Human MSCs were maintained in a 10% CO_2_ environment at 37 °C and were cultured in DMEM (Gibco) with 20% FBS (Gibco) and 10% penicillin (Gibco).

### Secretome preparation

After 10 days of neuroepithelial differentiation from iPSCs, 8 days of NSC differentiation, and subsequent CNSC differentiation by the previously described cell culture method, the culture medium was changed every 48 h, and the culture supernatants of the last 6 days were used as the material for CNSC-SE. For MSC-SE, the same conditions as that for CNSC-SE were used; cells were seeded and culture supernatant was collected after 3 days of culture. The collected culture supernatants were filtered through a 0.2-μm filter to remove cell debris. The filtered supernatants were stored at − 80 °C for 3 days, and the frozen samples were lyophilized for 7 days. The solid samples were stored at − 20 °C.

### Immunocytochemical analysis

iPSCs and differentiated cells (iPSC-derived CNSCs) used for in-vitro experiments were fixed with 4% paraformaldehyde (PFA), permeabilized with 0.1% Triton X-100, and blocked with 2% bovine serum albumin (BSA) for 40 min, before being incubated overnight with primary antibodies. The primary antibodies used are given in Table 1. Secondary antibodies were then treated for 1 h. DAPI was used for nuclear staining. Confocal microscopy was performed on LSM900 w/Airyscan (Carl Zeiss, Oberkochen, Baden-Württemberg, Germany). Images represent confocal Z-stack taken with identical laser and detection settings.

**Table 1:** List of antibodies used for immunocytochemistry

Antibody	Company	Catalog
BDNF	Abcam	ab108319
GDNF	Santa cruz	Sc-13147
VEGF	Santa cruz	Sc-7269
MAP-2	Santa cruz	Sc-74421
NEUN	Abcam	Ab104224
vGLUT	Synaptic system	135,303
Tuj1	Gentex	GTX631836
FOXG1	Abcam	Ab29359
Tbr1	Abcam	Ab31940
SATB2	Abcam	Ab51502
CUX1	Santa Cruz	Sc-514008
GFAP	Abcam	Ab7260
anti-Iba1	Fujifilm	#019–19741
6E10	BioLegend	803,001

*BDNF* Brain-derived neurotrophic factor, *GDNF* Glial cell-derived neurotrophic factor, *VEGF* Vascular endothelial growth factor, *MAP2* Microtubule associated protein 2, *NEUN* Neuronal nuclei, *vGLUT* Vesicular glutamate transporter, *GFAP* Glial fibrillary acidic protein, *CUX1* CUT-Like Homeobox 1, *SATB2* Special AT-rich sequence-binding protein 2

### Multielectrode array recording

The iPSC-derived cortical neurons were treated with 130 μg/ml CNSC-SE for 20 days. After treatment, $1.3\times {10}^{7}$ cells were seeded on a poly-*L*-ornithine/laminin-coated CytoView 24-well plate (Axion Biosystems, Atlanta, GA; #M384-tMEA-24W), and iPSCs and non-treated (0 μg/ml group) iPSC-derived cortical neurons were used as controls.

The AxIS software was used to measure the spontaneous potential activity of cortical neurons. Cells were seeded at a density of 1.3 × 10^7^ per well in a CytoView 24-well plate (Axion Biosystems, #M384-tMEA-24W) and cultured in 5% CO_2_ at 37 °C. The spontaneous activity was recorded for 15 min at the same time every day, and temperatures were maintained during all experiments.

### Real-time polymerase chain reaction (PCR)

RNA was extracted with Trizol reagent (Invitrogen, Waltham, MA). The RevertAid First strand cDNA Synthesis kit (Invitrogen, #K1622) was used for cDNA synthesis. Real-time PCR was performed with a PowerSYBR Green PCR Master Mix (Applied Biosystems, Waltham, MA; #436759) using the Real-Time PCR System (Applied Biosystems). The primer sequences are shown in Table 2.

**Table 2:** Primer sequences

Gene name		Sequence (5′→3′)	Length (bp)
BDNF	F	CGGAAGGACCTATGTTTGCT	106
	R	TATTTCAGAACGCGCAACTG	
GDNF	F	AGCTGCCAACCCAGAGAAT T	87
	R	AAATGTATTGCAGTTAAGACACAACC	
VEGF	F	CATTGGAGCCTTGCCTTG	87
	R	ATGATTCTGCCCTCCTCCTT	
NEUN	F	GCGGCTAACGTCTCCAACAT	188
	R	ATCGTCCCATTCAGCTTCTCCC	
vGlut	F	CCATGACTAAGCACAAGACTC	81
	R	AGATGACACCTCCATAGTGC	
MAP2	F	GGAGACAGAGATGAGAATTCC	82
	R	GAATTGGCTCTGACCTGGT	
Nestin	F	ACCAAGAGACATTCAGACTCC	303
	R	CCTCATCCTCATTTTCCACTCC	
PAX6	F	GTGTCCAACGGATGTGGAG	254
	R	CTAGCCAGGTTGCGAAAGAAC	
Tbr1	F	GGGCTCACTGGATGCGCCAAG	157
	R	TCCGTGCCGTCCTCGTTCACT	
TUJ1	F	GGCCTTTGGACATCTCTTCA	241
	R	ATACTCCTCACGCACCTTGC	
FOXG1	F	AGGAGGGCGAGAAGAAGAAC	213
	R	TCACGAAGCACTTGTTGAGG	
OCT4	F	ACCCCTGGTGCCGTGAA	190
	R	GGCTGAATACCTTCCCAAATA	
GAPDH	F	ACCCACTCCTCCACCTTTGA	101
	R	CTGTTGCTGTAGCCAAATTCGT	
mIGFBP-2	F	CAGACGCTACGCTG-CTATCC	142
	R	CTCCCTCAGAGTGGTCGTCA	
mIGF-2	F	TGGCCCTCCTGGAGACGTACTGTGC	116
	R	TTGGAAGAACTTGCCCACGGGGTATC	
mIGF-1r	F	CTACCTCCCTCTCTGGGAATG	185
	R	GCCCAACCTGCTGTTATTTCT	
mGAPDH	F	GCCAAACGGGTCATCATCTC	377
	R	GACACATTGGGGGTAGGAAC	

### Western blot

The whole brain of each mouse was homogenized with a protein extraction reagent (Thermo Fisher Scientific, Waltham, MA) supplemented with 1 protease inhibitor cocktail tablet (Roche, Basel, Switzerland) and 1 mM phenylmethylsulfonyl fluoride. The homogenate was sonicated and centrifuged at 4 °C for 20 min at 16,000 rpm. Proteins were quantified using the Bradford dye (Bio-Rad, Hercules, CA) with BSA as a standard. Next, 30 μg of total proteins was separated by sodium dodecyl sulphate–polyacrylamide gel electrophoresis and transferred to a nitrocellulose membrane. The membrane was blocked with 3% BSA for 1 h at room temperature (RT) and incubated at 4 °C overnight with primary antibodies (Table 3). After washing with TBST, the membrane was incubated with the secondary antibody for 1 h at RT. Protein bands were visualized using enhanced chemiluminescence. Band intensities were normalized to β-actin.

**Table 3:** List of primary antibodies used for Western blotting

Antibody	Company	Catalog No.	Dilution for Western blotting
anti-APP	Millipore	A8717	1:1000
anti-pTau	Cell signaling	D4H7E	1:500
BACE	Cell signaling	D10E5	1:1000
anti-Iba1	Fujifilm	019-19741	1:500
anti-TNFα	Abcam	Ab9739	1:500
anti-IL-1β	Abcam	Ab9722	1:500
GAPDH	Abcam	Ab9485	1:1000

### Immunohistochemistry for Aβ plaque

Mice were intracardially perfused with saline, and their brains were collected and fixed in 4% PFA at 4 °C, followed by dehydration in 15% sucrose and then 30% sucrose. The brains were then embedded in OCT Compound (Sakura Finetek, Torrance, CA) and sectioned (9 μm) with a microtome (Thermo Fisher Scientific) onto gelatin-coated slides (Masterflex, Gelsenkirchen, Germany; #HV-75955-51). These brain section slides were stored at − 80 °C.

For immunohistochemistry, the slides were dried overnight at RT and fixed with cold acetone for 10 min. Endogenous peroxidase activity was blocked by treating all slides with 0.3% H_2_O_2_. The slides were incubated for 1 h with 10% normal goat serum and thereafter overnight at 4 °C with anti-Aβ (1:500, Abcam, Cambridge, UK, #ab201060). After incubation with biotinylated secondary antibodies (1:200) for 40 min, the slides were incubated with the ABC HRP reagent (Vector Laboratories, Newark, CA; #PK-7100) for 10 min and DAB (Vector Laboratories) for 1 min. The slides were counterstained with Mayer’s hematoxylin.

### Immunofluorescence analysis of mouse brain tissue

The brain sections were dried overnight at RT and fixed with cold acetone for 10 min. Endogenous peroxidase activity was blocked by treating the slides with 0.3% H_2_O_2_. Then the sections were blocked with Mouse on Mouse (M.O.M.) IgG blocking reagent (M.O.M. Fluorescein Kit, Vector Laboratories, #FMK-2201) diluted in 1% BSA for 5 min, followed by incubation overnight at 4 °C with 6E10 (1:1000, BioLegend, #803001). The next day, the slides were incubated with M.O.M. Biotinylated anti-mouse IgG and with the secondary antibody attached to fluorescein Avidin DCS.

For GFAP and IBa-1 immunofluorescence staining, the sections were incubated with anti-GFAP (1:1000, Abcam, #ab7260) or anti-IBa-1 (1:1000, Fujifilm, #019-19741) in 10% normal goat serum at 4 °C overnight. Then the sections were incubated with secondary antibodies such as goat anti-rabbit IgG Alexa 594 (1:200, Thermo Fisher Scienctific) for 1 h. Confocal microscopy was performed on LSM900 w/Airyscan, Carl Zeiss. Confocal Z-stack images were taken with identical laser and detection settings.

### Cresyl violet staining

The brain sections were dried overnight at RT, fixed with cold acetone for 10 min, rehydrated in 70% and 95% alcohol for 2 min each, and stained with cresyl violet solution (Abcam, ab246816) for 1 min. Then they were washed in 70% and 95% alcohol for 2 min each, dehydrated in 100% ethanol for 30 s, cleared with xylene twice and mounted.

### Metabolomics analysis

Metabolomics analysis for iPSC secretomes and CNSC-SE was performed using capillary electrophoresis mass spectrometry (Human Metabolome Technologies, Inc, Yamagata, Japan). Samples were prepared to allow for metabolite extraction. To perform metabolomics analysis, we used three samples of iPSC secretomes and CNSC-SE each, and Essential 8 media and neural basal media were used as a control.

### Human cytokine array

Cytokines in CNSC-SE were assayed using a human XL cytokine array kit (ARY022B, R&D Systems, Minneapolis, MN) according to the manufacturer's protocol. The membranes of the kit were blocked at RT for 1 h, and then incubated with culture medium overnight at 4 °C. After 3 days of culture, the medium was debris removed with a 0.22 um syringe filter and used for experiments. The detection antibody cocktail diluted in the assay buffer was added and incubated at 25 °C for 1 h, and 1 × Streptavidin-HRP was added and incubated at RT for 30 min. All experiments were performed on a shaker. Images were acquired using a bio-image analysis system (Amersham Imager 600), and quantitative evaluation was performed using the Image J software.

### Growth factor array

The human growth factor antibody array (ab134002; Abcam) was performed according to the manufacturer’s protocol. The lyophilized CNSC-SE and MSC-SE samples used in the experiments were diluted to 0.1 g/ml in blocking buffer. Membranes blocked at RT for 1 h were incubated with samples overnight at 4 °C. The biotin-conjugated anti-cytokines cocktail, diluted 1000 × , was added and incubated for 2 h. After washing, HRP-Conjugated Streptavidin was added and incubated for 1 h at RT. The membrane was then exposed using a bio-image analysis system (Amersham Imager 600).

### Statistical analysis

All experiments were repeated at least three times, and results are expressed as the mean and standard error of the mean (shown as error bars). Statistical analysis was performed using GraphPad Prism 9.0. Comparison of multiple groups was performed by one-way analysis of variance (ANOVA) followed by *post-hoc* Dunnett’s multiple comparison test. A *t*-test was used to analyze nonparametric quantitative datasets, and one-tailed* P* values were calculated. Differences between groups were examined for statistical significance using Welch’s *t*-test, which was also used for analyzing metabolomics. Kruskal–Wallis and Mann–Whitney analyses were performed for intergroup comparison. Statistical significance was set at *P* < 0.05.

## Results

### iPSC-derived CNSC-SE promotes cortical neuron differentiation in vitro

**Fig. 1:**
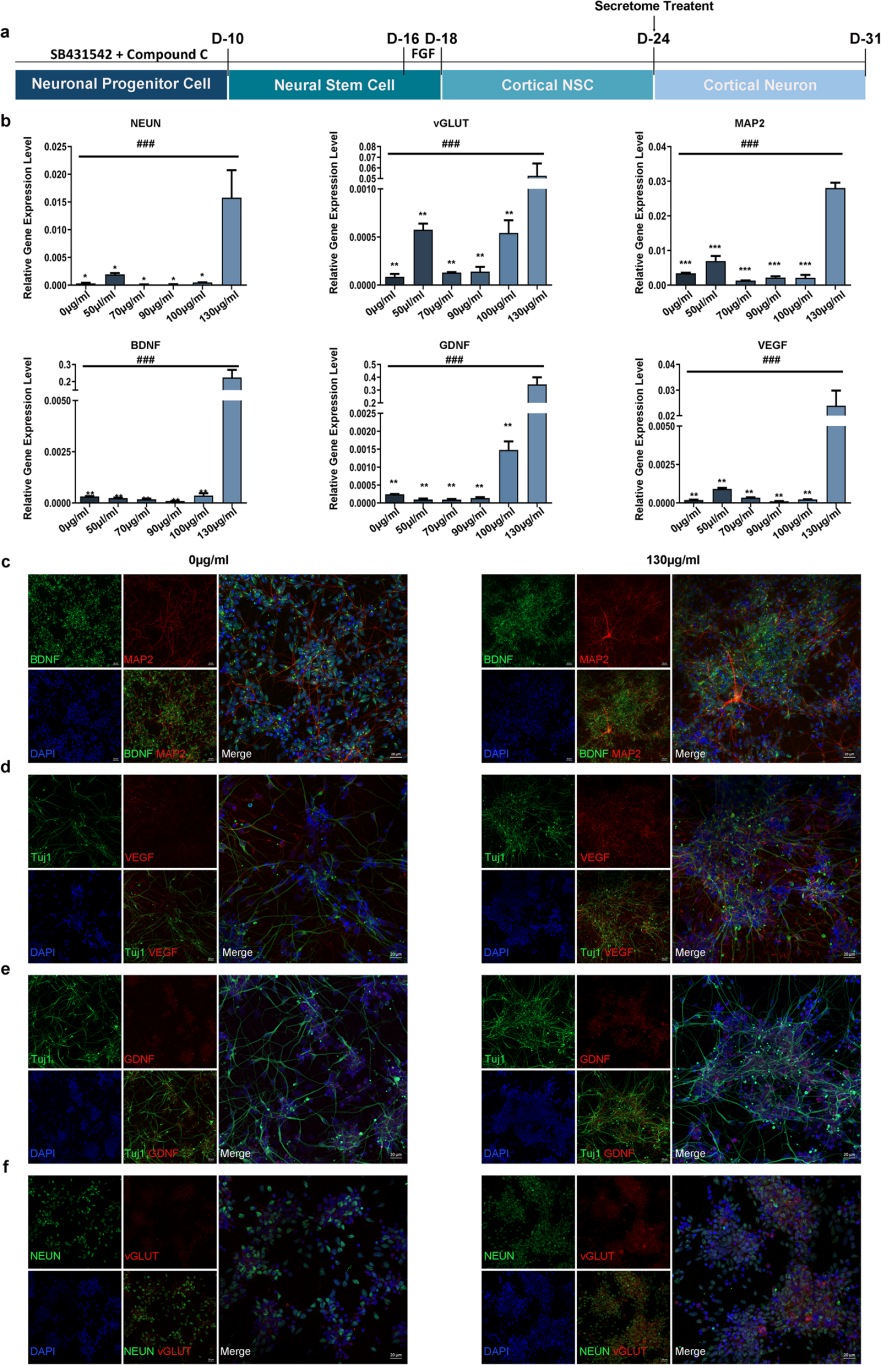
iPSC-derived CNSC-SE promoted cortical neuron differentiation. **a** Schematic of treatment schedule. **b** Relative expression of neural markers (NEUN, vGLUT, and MAP2) and growth factor genes (BDNF, GDNF, and VEGF) in cells treated with various concentration conditions (0 μg/ml, 50 μg/ml, 70 μg/ml, 90 μg/ml, 100 μg/ml, and 130 μg/ml). **c**–**f** Immunofluorescence staining of neural markers (MAP2, Tuj1, NEUN, and VGLUT1) and growth factors (BDNF, GDNF, and VEGF) in cells treated with 0 or 130 μg/ml CNSC-SE. Data are presented as mean ± SEM. One-way ANOVA; *P < 0.05; **P < 0.01; ***P < 0.001, Dunnett’s test. BDNF, brain-derived neurotrophic factor; GDNF, glial cell-derived neurotrophic factor; VEGF, vascular endothelial growth factor; NEUN, neuronal nuclei; vGLUT, vesicular glutamate transporter; MAP2, microtubule-associated protein 2; iPSC, induced pluripotent stem cells; CNSC-SE, cortical neural stem cell secretome

In this study, we used the iPSC-derived cortical neuron differentiation protocol based on a previously reported study (Additional file 1: Fig. S1a) [37]. The cortical identity of the neural tissue formed was validated by the expression of genes expressed in cortical neuronal cells during differentiation and by the absence of expression of other regionally expressed genes (Additional file 1: Fig. S1b). The protein expression of specific markers was confirmed by immunofluorescence staining (Additional file 1: Fig. S1c). To determine if CNSC-SE promotes cortical neuron differentiation, CNSC-SE was treated at a range of concentrations (0 μg/ml, 50 μg/ml, 70 μg/ml, 90 μg/ml, 100 μg/ml, and 130 μg/ml) over a period of 7 days (Fig. 1a). The cortical neurons were confirmed with the expression of neural markers, such as neuronal nuclei (NEUN), vesicular glutamate transporter (vGLUT), and microtubule-associated protein 2 (MAP2), and growth factors, such as brain-derived neurotrophic factor (BDNF), glial cell-derived neurotrophic factor (GDNF), and vascular endothelial growth factor (VEGF). In cortical neurons treated with 130 μg/ml CNSC-SE, the mRNA expression of neural markers and growth factors was significantly increased compared to that of other concentrations (Fig. 1b). Therefore, this concentration was selected for in vitro administration.

In the immunofluorescence assay, the 130 μg/ml CNSC-SE-treated cortical neurons showed higher expression of markers and growth factors compared to the cortical neurons treated with 0 μg/ml, such as MAP2, which is a predominant cytoskeletal regulator within neuronal dendrites (Fig. 1c), BDNF, VEGF (Fig. 1d–e), and vGLUT, a marker of mature neurons (Fig. 1f). Taken together, we confirmed that the iPSC-derived CNSC-SE increased neuronal differentiation in vitro and possibly improved dendritic structure by increasing the expression of MAP2.

### Cortical neurons treated with CNSC-SE acquire electrical network activity and action potential bursts

**Fig. 2:**
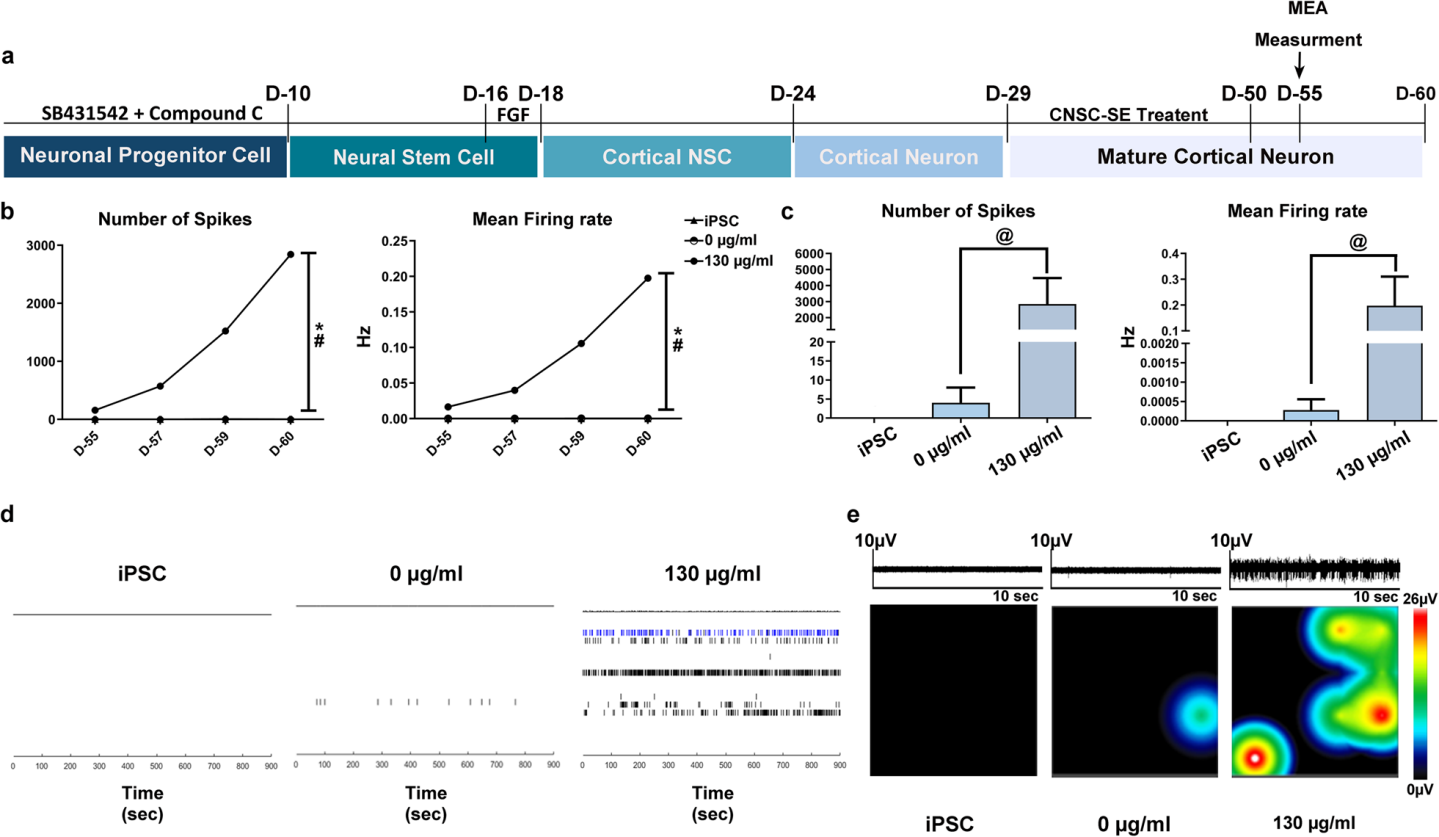
iPSC-derived CNSC-SE increased neuronal network activity and action potential bursts. **a** Overview of secretome treatment and MEA schedules. **b** MEA analysis of cortical neurons administered with CNSC-SE from day 50 to day 60. **c** MEA measurement on day 60. **d** Raster image of neuronal network activity over a period of 900 s. **e** Representative electrical activity of cortical neurons administered with CNSC-SE. As controls, iPSCs and cortical neurons were evaluated. Data are presented as mean ± SEM. One-way ANOVA; ^*#^*P* < 0.05 (*iPSC *vs.* 130 μg/ml, #0 μg/ml *vs.* 130 μg/ml), Dunnett’s test. Kruskal–Wallis and Mann–Whitney analyses were performed for intergroup comparison; ^@^*P* < 0.05. Abbreviations: MEA, multiwell microelectrode array; iPSC, induced pluripotent stem cell; CNSC-SE, cortical neural stem cell secretome

iPSC-derived mature cortical neurons were treated with CNSC-SE once every 2 days, for 20 days, to test the effects of CNSC-SE on electrical network activity. After 20 days of treatment, the cells were attached to a multiwell microelectrode array (MEA) plate, and MEA was measured for a week from day 55 (Fig. 2a). Interestingly, the electromagnetic signal was detected in the CNSC-SE-treated cortical neurons from the first day of attachment. The electrical signals increased gradually over time, suggesting that the CNSC-SE-treated neurons acquired synchronous network activity (Fig. 2b). The spontaneous activity at day 60 was significantly increased in the CNSC-SE-treated cortical neurons (Fig. 2c–e), especially in the roaster plot, showing a single-channel burst (blue line) pattern (Fig. 2d). Heat map activity showed that the CNSC-SE-treated cortical neurons had increased spike density and area within the cortical networks (Fig. 2e). The iPSC-derived cortical neuron differentiation protocol was reported to exhibit mature electrical signals on day 80 after differentiation [37]; however, in our study, the CNSC-SE-treated cortical neurons showed electrical signals on day 55, confirming that CNSC-SE had potential effects on the development of electrical network.

### Intranasal delivery of iPSC-derived CNSC-SE ameliorates spatial memory and cognitive impairments in 5×FAD mice

**Fig. 3:**
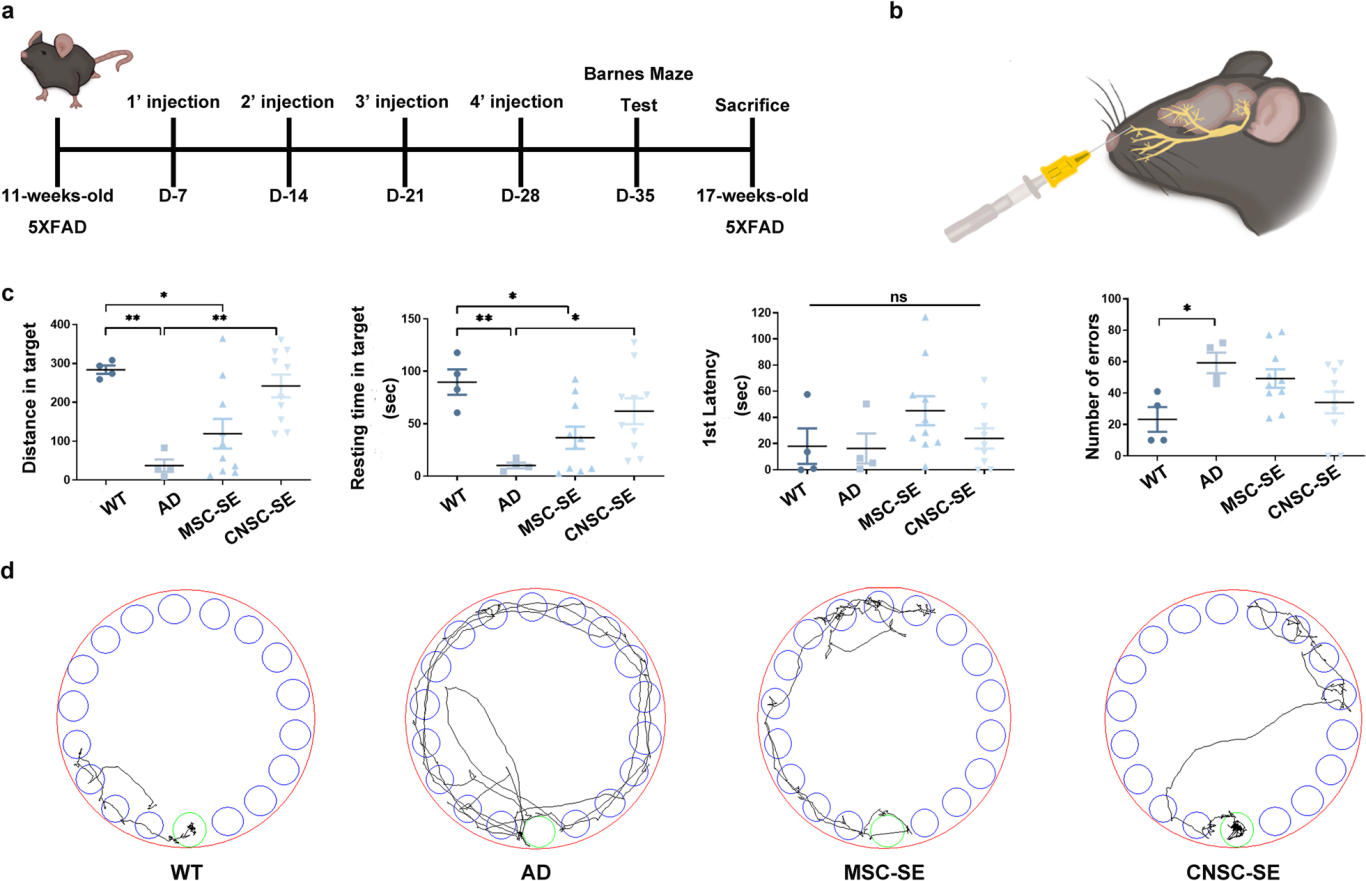
Intranasal delivery of iPSC-derived CNSC-SE significantly improved memory in AD mice. **a** Schematic image of injection schedule. **b** Intranasal delivery design: (1) wild-type mice as controls (WT group); (2) 5×FAD AD model (AD group, *n* = 4); (3) CNSC-SE-treated 5×FAD mice (*n* = 10); and (4) MSC-treated 5×FAD mice (*n* = 10). **c** Monitoring of improved memory by Barnes maze. **d** Track plots. Data are presented as mean ± SEM. One-way ANOVA; *P < 0.05; **P < 0.01; ***P < 0.001, Dunnett’s test. iPSC, induced pluripotent stem cells; MSC, mesenchymal stem cells; CNSC-SE, cortical neural stem cell secretome

The ultimate goal of AD treatment is to improve memory. For more efficient CNSC-SE delivery, we used the intranasal route, which is non-invasive and provides the benefits of brain delivery via the olfactory pathway. In addition, to confirm the efficiency of CNSC-SE as a targeted treatment for AD, MSCs, which are known to be specifically effective in neuro-inflammatory processes and in memory recovery, were used as a comparison group. The 12-week-old 5×FAD mice were administered with the secretome intranasally at the dose of 5 μg/g body weight (Additional File 2: Fig. S2) once a week, for 4 weeks, and were tested 1 week later in the Barnes maze (Fig. 3a, b). The CNSC-SE-treated group showed significant improvement in behavioral performance based on the measurements of the following parameters: distance from target zone, resting time in target zone, and error rate. In particular, similar to the control group (WT), mice in the CNSC-SE-5×FAD group stayed in the target hole for a significantly longer time compared to the AD group (Fig. 3c). There was no significant difference between the two groups with respect to the first latency. In addition, regarding the error rate, the CNSC-SE-5×FAD mice showed the next lowest error rate after WT (Fig. 3c). The distinct efficacy of CNSC-SE treatment in improving cognition and memory recovery can also be seen from the tracks (Fig. 3d) and the videos (Additional files 2, 3, 4, and 5). The 3-min camera tracking system showed that the AD model mice did not find the target zone easily. On the contrary, the CNSC-SE-5×FAD mice showed a similar pattern to that of the WT mice and had fewer erroneous entries than the MSC-SE-5×FAD mice. These results suggest that intranasal injection of CNSC-SE may ameliorate cognitive impairment and increase spatial cognition of AD mice.

### Intranasal delivery of iPSC-derived CNSC-SE reduces amyloidosis and neuro-inflammatory proteins in 5×FAD mouse brain

**Fig. 4:**
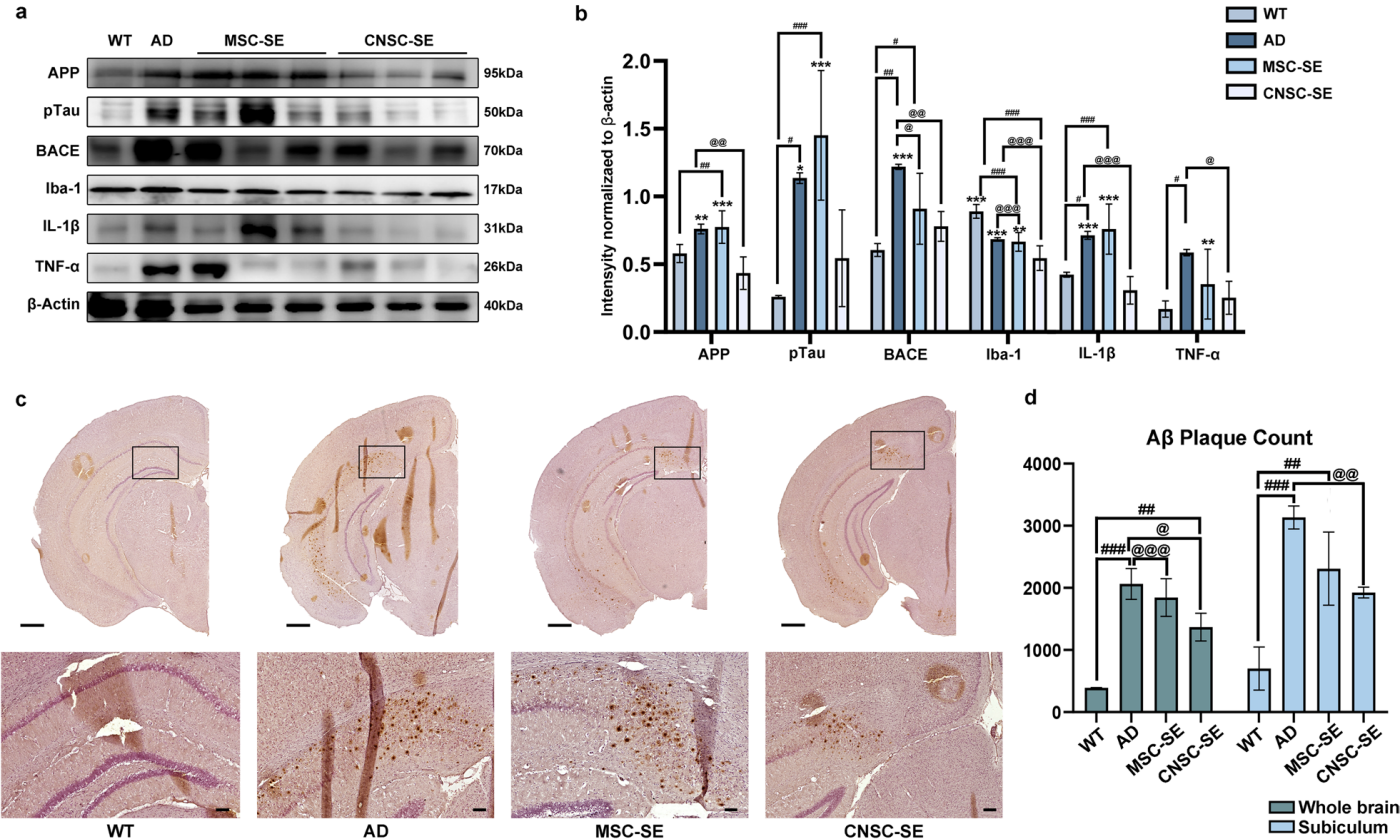
Intranasal delivery of iPSC-derived CNSC-SE reduced expression of AD-related proteins in AD mouse brain. **a** Expression of protein markers involved in AD (APP, pTau, and BACE), inflammatory cytokines (TNFα and IL-1β), and neuroinflammatory markers (Iba-1 and GFAP) in the mouse whole brain. **b** Quantification of the proteins, normalized to β-actin. **c** Brain sections were stained using immunohistochemistry with Aβ monoclonal antibody in the following groups: (1) wild-type mice as controls (WT group); (2) 5×FAD AD model (AD group); (3) CNSC-SE-treated 5×FAD mice; and (4) MSC-treated 5×FAD mice. **d** Amyloid plaques were counted in the whole brain and in the subiculum using Image J (measured three times with the same place). Scale bars, 200 μm. Data are presented as mean ± SEM. *P < 0.05; **P < 0.01; ***P < 0.001 *vs.* CNSC-SE (T-rest), ^#^*P* < 0.05, ^##^*P* < 0.01, ^##^*P* < 0.001 *vs.* WT (One-way ANOVA), ^@^*P* < 0.05, ^@@^*P* < 0.01, ^@@@^*P* < 0.001 *vs.* AD (One-way ANOVA). MSC, mesenchymal stem cells; CNSC-SE, cortical neural stem cell secretome; GFAP, glial fibrillary acidic protein; TNFα, tumor necrosis factor α

To investigate the anti-amyloid and anti-inflammatory effects of CNSC-SE in AD, we performed Western blotting for AD-specific markers in mouse brain. Amyloid precursor protein (APP) was significantly decreased in CNSC-SE-5×FAD mouse brains compared to the AD group (Fig. 4a, b). The expression of phosphorylated tau (pTau) was significantly higher in AD and MSC-SE-5×FAD compared to WT, while CNSC-SE-5×FAD did not show any significant difference from WT. We found that beta-site APP cleaving enzyme (BACE), which is an enzyme essential for Aβ generation, was significantly reduced in the CNSC-SE-5×FAD mice (Fig. 4a, b). These results demonstrated a potential protective effect of CNSC-SE in modulating the rate of AD progression, not only by reducing the expression of Aβ precursor APP, but also by regulating the expression of BACE, which initiates Aβ production. To confirm the effect of CNSC-SE on the excessive immune-inflammatory response following amyloid plaque infiltration in AD brain, we investigated the expression of Iba-1, a specific marker for microglial cells (Fig. 4a, b). The expression of Iba-1 was reduced in the secretome-treated groups (CNSC-SE and MSC-SE) compared to the AD model group. Interestingly, a significant reduction of Iba-1 was seen in the CNSC-SE-5×FAD mice compared to both WT and AD (Fig. 4b). Expression of interleukin-1β (IL-1β) and tumor necrosis factor-α (TNFα) was decreased in the 5×FAD mouse brains treated with secretomes (CNSC-SE and MSC-SE). Immunohistochemistry showed pronounced plaque deposition in AD mouse brain, especially in the subiculum location, whereas significantly less accumulation was found in the CNSC-SE-5×FAD mice compared to the AD mice (Fig. 4c, d). When we further checked plaque deposition in the ventral hippocampus, we found a trend of plaque reduction in the CNSC-SE mice compared to the AD and the MSC-SE mice, although there was no significant difference (Additional file 1: Fig. S3).

**Fig. 5:**
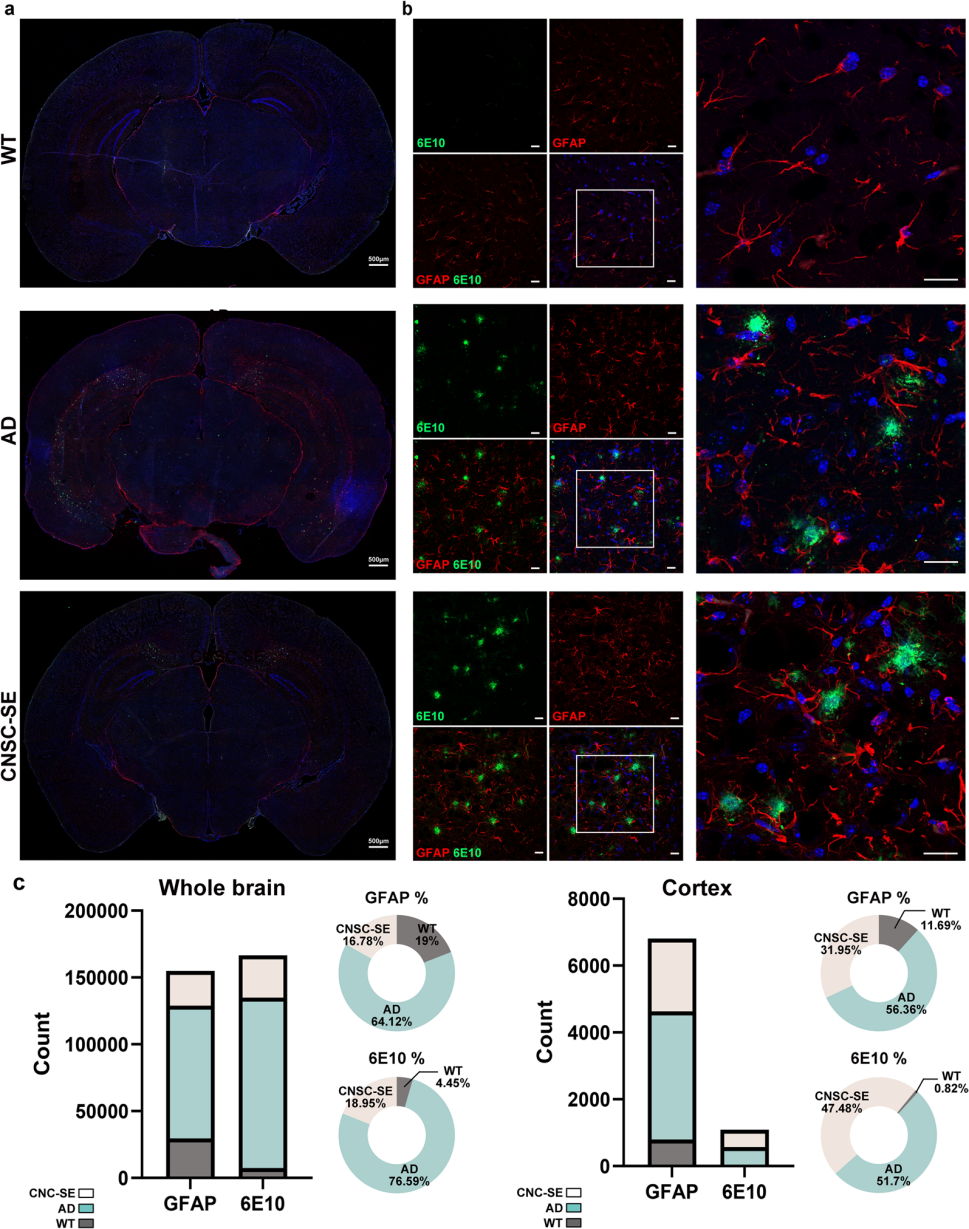
Intranasal delivery of iPSC-derived CNSC-SE improved the excessive anti-inflammatory response. **a** Confocal images of GFAP immunostaining in the whole brains of 5×FAD (AD model mice) and CNSC-SE-treated 5×FAD mice. **b** Representative images of Aβ (6E10) plaques and astrocyte (GFAP) co-staining in the cortex of 5×FAD mice and CNSC-SE-treated 5×FAD mice. **c** Quantification of GFAP-positive astrocytes and internalized Aβ using the Image J software. Scale bars, 20 μm. iPSC, induced pluripotent stem cells; CNSC-SE, cortical neural stem cell secretome; GFAP, glial fibrillary acidic protein

In AD, activated glial cells promote the removal of amyloid plaques while simultaneously causing excessive neurotoxicity and memory impairment. In Fig. 5a and c, the number of plaques in the AD brain was greater than that of CNSC-SE-5×FAD mice. By quantifying the plaques in the whole brain, we also confirmed that the expression of glial fibrillary acidic protein (GFAP), an astrocyte marker, was three times higher in AD mouse brain (Fig. 5c). Astrocytes in AD are known to have reduced expression of synaptic proteins, suggesting an excessive immune response, in contrast to healthy astrocytes that protect against synapse loss [38, 39]. While the morphology of astrocytes in the CNSC-SE-5×FAD mouse brain was not that different from AD mice brain (Fig. 5b), the number of GFAP-positive cells was significantly decreased in the whole brain as well as in the cortex (Fig. 5c).

**Fig. 6:**
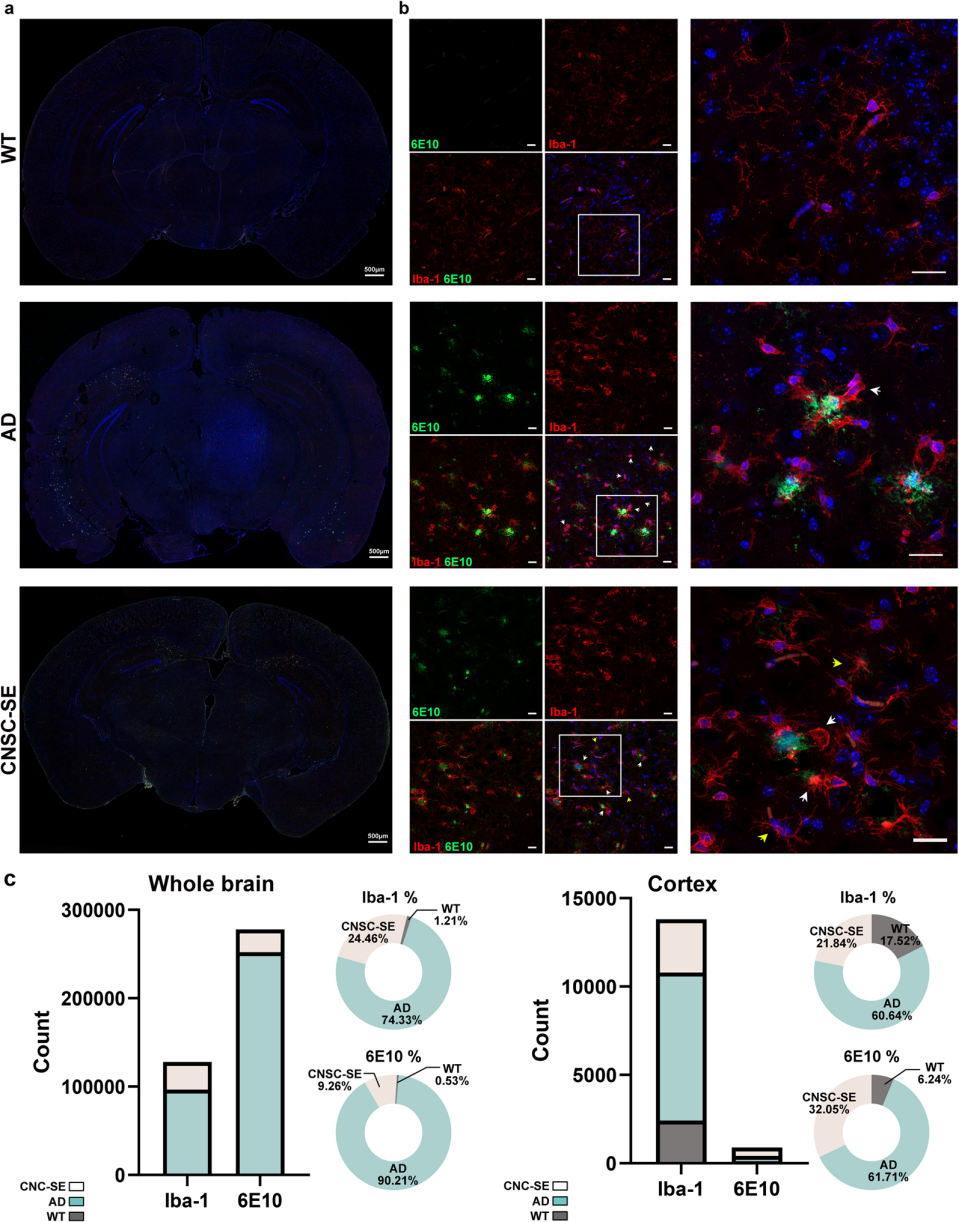
Anti-inflammatory effects of CNSC-SE on hyperexcitable microglia. **a** Confocal images of Iba-1 immunostaining in the whole brains of 5×FAD (AD model mice) and CNSC-SE-treated 5×FAD mice. **b** Representative images of Aβ (6E10) plaques and microglia (Iba-1) co-staining in the cortex of 5×FAD mice and CNSC-SE-treated 5×FAD mice. **c** Quantification of Iba-1-positive microglia and internalized Aβ using the Image J software. Scale bars, 20 μm

To determine the extent of microglial and astrocyte activation, we characterized their morphology and distribution by fluorescent staining and quantified their numbers (Figs. 5 and 6). Microglia in AD brains exposed to high inflammatory conditions are known to have enhanced phagocytosis, accelerating the progression of the disease [40]. Therefore, we also assessed the degree of inflammatory activation of microglia (Iba-1) (Fig. 6). In the AD mouse brain, many round-shaped activated microglia (Fig. 6b, white arrow) were observed especially around the amyloid plaques, whereas in the CNSC-SE mouse brain, dormant microglia (yellow arrows) could be found, and only microglia around the plaques were activated (Fig. 6b). These results suggest that intranasal administration of CNSC-SE potentially reduced the activation of abnormal microglia. Taken together, our results suggest that CNSC-SE treatment not only reduced the deposition of amyloid plaques in the AD brain, but also reduced the excessive inflammatory response by inhibiting the abnormal activation of glial cells.

### IGF-binding protein 2 (IGFBP-2) is a possible therapeutic candidate in the iPSC-derived CNSC-SE

**Fig. 7:**
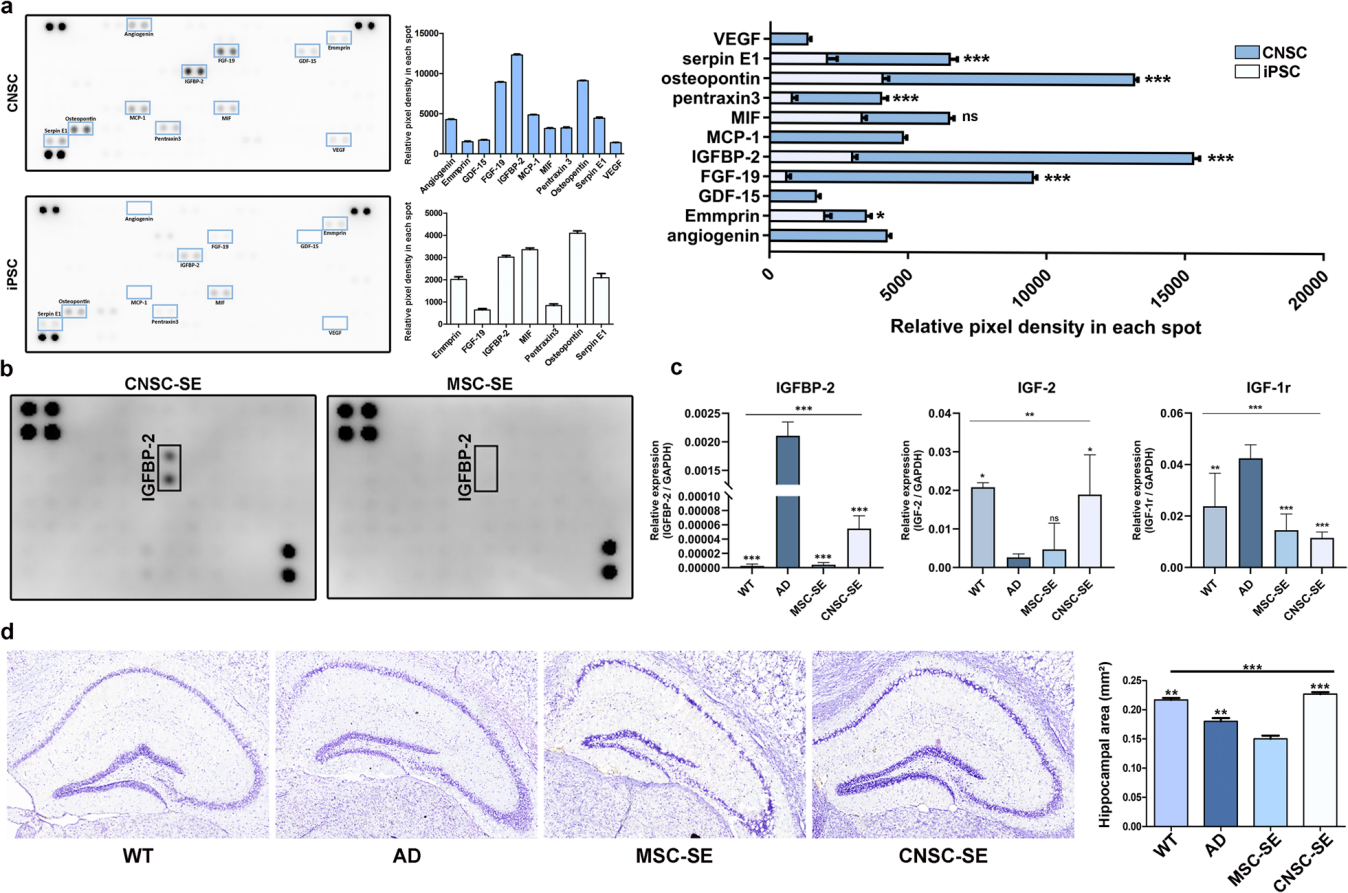
Cytokine and growth factor analysis of iPSC-derived CNSC-SE. **a** Human cytokine array images of CNSC and iPSC conditioning media and quantification. **b** Human growth factor array images for CNSC-SE and MSC-SE. **c** Relative expression of IGFBP-2, IGF-2 and IGF-1r in 5×FAD brain. **d** Quantitative assessment of hippocampal area by Cresyl violet staining. Data are presented as mean ± SEM. *P < 0.05, **P < 0.01, ***P < 0.001 (T-test). ^*^P < 0.05, ^**^P < 0.01, ^***^P < 0.001 *vs.* AD (One-way ANOVA)

To identify the possible cytokines and growth factors in the CNSC-SE, we performed a cytokine array using the conditioned medium of CNSCs and iPSCs collected on day 3 of culture (Fig. 7a). We identified 11 cytokines in the conditioned medium of CNSCs and 7 cytokines in that of iPSCs. Most cytokines were highly expressed in CNSCs, especially IGFBP-2, FGF-19, and osteopontin, which were significantly higher than those in iPSC medium. IGFBP-2 showed a fourfold increase in the CNSC medium compared to that in the iPSC medium. We also performed a human growth factor array for CNSC-SE and MSC-SE. Interestingly, only IGFBP-2 in CNSC-SE was confirmed, while no signal was expressed in MSC-SE (Fig. 7b). These results indicate that CNSC-conditioned media and CNSC-SE uniquely retain IGFBP-2.

IGFBP-2 is a polypeptide that is most abundant in the cerebrospinal fluid, the developing brain, the hippocampus and the cortex, and functions as an autocrine and/or paracrine growth factor. While insulin-like growth factor 2 (IGF-2) can be regulated by IGFBP-2, only IGF-2 expression correlated with the expression of IGFBP-2 throughout the central nervous system (CNS) development, suggesting that IGFBP-2 may regulate the function of IGF-2 in the CNS [41]. Here, we confirmed the expression of IGFBP-2 and IGF2/IGF-1r in SE-treated mouse brains (Fig. 7c). IGFBP-2, a neuronal proliferation growth factor responsible for cognition and information processing in the brain, was highly expressed in AD and was about three times higher in CNSC-SE treated mice brain compared to trace amounts in MSC-SE-treated mice. IGF-2 has been reported to be decreased in AD patients and mouse models, and amyloid plaques are reduced in the hippocampus of transgenic mice overexpressing IGF-2 [42, 43]. Here, IGF-2, which is known to improve brain function and reduce amyloid plaques, was significantly increased in CNSC-SE-5×FAD compared to AD (Fig. 7c). IGF-1r, on the other hand, showed the highest expression in AD and was significantly reduced in MSC-SE-5×FAD and CNSC-SE-5×FAD (Fig. 7c). IGF-1r is involved in several key biological pathways related to the aging process, and IGF-2 binds to IGF-1r with high affinity [44]. It is reported that IGF-1r is involved in disease exacerbation [45]. However, recent studies have reported that IGFBP-2 is highly expressed in the serum of AD patients, and higher IGFBP-2 levels are only observed in amyloid-negative individuals with smaller hippocampal volumes. Therefore, cresyl violet staining was performed to measure the size of the hippocampus (Fig. 7d). We found an average hippocampal area of 0.23 mm^2^ in WT and CNSC-SE-5×FAD. In addition, the hippocampus of the 5×FAD mice was not shrunk after CNSC-SE treatment. These results suggest that CNSC-SE, which contains high IGFBP-2, functions as a paracrine growth factor to repair the AD brain by promoting IGF-2 and reducing IGF-1r in the AD environment.

### Qualitative metabolomics of iPSC-derived CNSC-SE

**Fig. 8:**
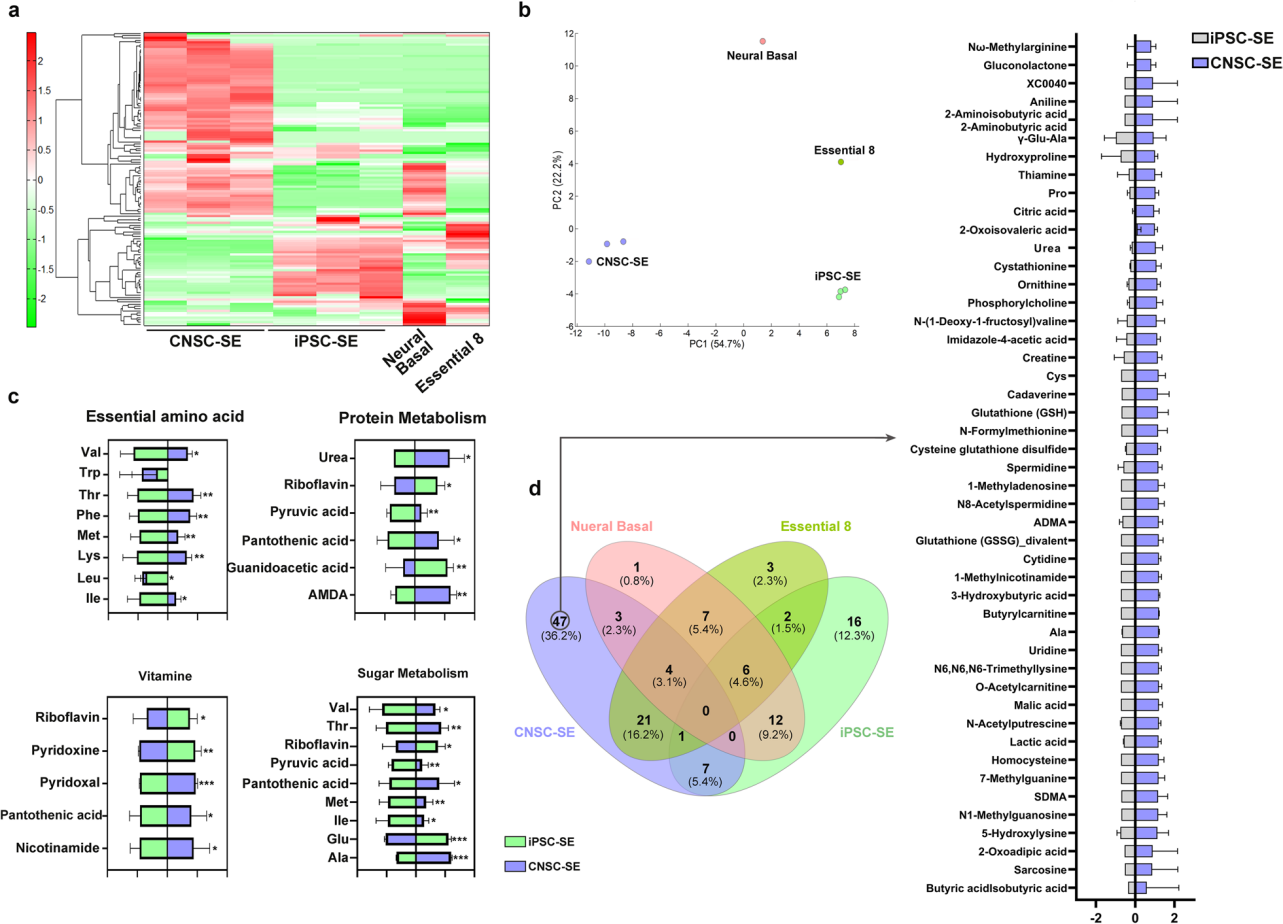
Metabolomics analysis of iPSC-derived CNSC-SE. **a** HCA was performed on peaks, and the distances between peaks are represented in tree diagrams. **b** PC1 and PC2 show the first principal component and second principal component respectively. The numbers in parentheses represent the contribution rate. **c** Comparative analysis of Human Metabolome Database physical property classification between CNSC-SE and iPSC-SE. **d** Venn diagram of total metabolomics compound from four batches. Data are presented as mean ± SEM. Welch’s T-test; *P < 0.05, **P < 0.01, ***P < 0.001. iPSC, induced pluripotent stem cells; CNSC-SE, cortical neural stem cell secretome; GFAP, glial fibrillary acidic protein; TNFα, tumor necrosis factor α

Metabolomics array was performed to determine which components of CNSC-SE promote neuronal differentiation and show disease-relieving effects in AD model mice [46]. As CNSC-SE was differentiated from iPSCs, the iPSC secretome (iPSC-SE) was used as a comparison group. In addition, the neural basal medium contained in the secretome was further analyzed. Comparison of the normalized semi-quantitative metabolite profiles of CNSC-SE with iPSC-SE yielded different trends (Fig. 8a). Comparison of the normalized semi-quantitative metabolite profile of CNSC-SE versus the neural basal medium by principal component analysis highlighted a clear difference between them, further demonstrating that the CNSC-SE and neural basal medium are biochemically distinct (Fig. 8b). Metabolites showing a significant difference between CNSC-SE and iPSC-SE were classified by the Human Metabolome Database physical property classification. In the essential amino acid classification, six metabolites, except “Trp”, were significantly present in CNSC-SE (Fig. 8c). In the vitamin metabolism classification, metabolites such as “nicotinamide,” which has antioxidant effect, were expressed to a higher extent in CNSC-SE (Fig. 8c). A total of 130 metabolites were identified. Interestingly, 47 metabolites were only detected in CNSC-SE (Fig. 8d), demonstrating the unique presence of many metabolites in CNSC-SE compared to iPSC-SE and neural basal medium. Among the 47 metabolites, sarcosine has been reported to improve behavioral defects by activating *N*-methyl-*D*-aspartate receptor (NMDAR)-induced electrophysiological activation in the NMDAR dysfunctional mouse model [47]; uridine is used as a component of Fortasyn Connect, a specially prepared drink that contains certain nutrients that are susceptible to deficiency in cognitive impairment and early dementia [48]. In addition, glutathione (GSH) is reported as a metabolic product that plays a major role as an antioxidant, an enzyme aid, and a neuromodulator in the CNS [49]. Cystathione is a rate-limiting step for GSH synthesis [50]. Creatine [51, 52] plays an important role in the energy homeostasis of neurons [53], and thiamine [54–56] plays an important coenzymatic role and is involved in biochemical reactions; both of them can improve brain function. Thus, it can be stated that the CNSC-SE-unique metabolites included several components that have various nerve-protecting functions or have confirmed effects of improving cognitive function recovery. In particular, GSH, which has antioxidant functions in the brain against oxidative stress, was also expressed higher than iPSC-SE alone.

## Discussion

AD is a progressive neurodegenerative disease characterized by memory loss and cognitive impairment, caused by synaptic disorders and excessive accumulation of incorrectly folded proteins [4, 34]. To date, almost all advanced clinical trials on specific AD-related pathways have failed due to the loss of large numbers of neurons in the brains of most AD patients [4, 13]. Stem cell-based treatments have emerged as new treatment strategies for various neurodegenerative diseases due to their self-renewability, versatility, and the ability to differentiate into major cell phenotypes in the CNS [15]. Recent preclinical studies of stem cell therapy for AD have proven promising; however, many hurdles exist for stem cell therapy to become a clinically feasible treatment for human AD and related diseases [3, 16].

Therefore, based on the report that the anti-amyloid efficacy of MSCs in AD is due to the side secretion effect [16], the secretome was separated from the iPSC-derived CNSC and used as a new AD-specific treatment candidate for this study. In addition, an intranasal administration method was used to efficiently deliver it to the brain in a non-invasive manner in consideration of elderly patients with AD.

Our main findings are as follows: (1) iPSC-derived cortical neurons facilitated neuroelectromagnetic signaling by increasing cortical neural network development and neuron maturation in vitro. (2) CNSC-SE treatment improved the memory of 5×FAD mice. (3) The number of Aβ plaques was decreased in the CNSC-SE-treated mice. This is thought to be potentially caused by a decrease in β-secretase, an enzyme that produces Aβ. (4) IGFBP-2 is the possible candidate responsible for the efficacy of iPSC-derived CNSC-SE in AD. (5) We also established a protocol for efficient delivery using intranasal administration via the olfactory nerve pathway without damaging the brain.

In this study, we successfully differentiated iPSCs into cortical neurons based on the previously reported protocol [37] and isolated CNSC-SE from the cell culture medium. In the process of cortical nerve differentiation of iPSCs, it was confirmed that nerve cells exposed to CNSC-SE showed dose-dependent increase of neural marker expression compared to those that were not exposed (Fig. 1f). Furthermore, electrical network activity, which is a major feature of neurodevelopment, was present in cortical neurons treated with CNSC-SE from day 55, confirming that CNSC-SE had a potential impact on electrical network development through increasing spike area and density (Fig. 2e).

One of the ultimate goals of AD treatment is memory recovery. Here, CNSC-SE was efficiently delivered to the brain of 5×FAD mice through a non-invasive route, and Barnes maze test was conducted to evaluate neuronal development, cognitive impairment, and spatial learning recovery. The 5×FAD mice treated with CNSC-SE showed memory improvement compared with those without CNSC-SE treatment (Fig. 3). In addition, histological analysis of brain samples showed that CNSC-SE treatment caused plaque reduction. The expression of neuroinflammatory factors and BACE (a β-secretase that promotes Aβ cleavage) was also decreased (Fig. 4). Compared to MSC-SE, CNSC-SE showed better efficiency in reducing APP and BACE. Taken together, the isolated CNSC-SE had similar effects to that of the MSC-SE, with several additional beneficial effects.

We also demonstrated that CNSC-SE reduced the inflammatory neuroimmunocyte phenotype by significantly reducing TNFα and IL-1β expression (Fig. 4b). In addition, the expression of inflammatory cytokines in the CNSC-SE group was significantly lower than that in the MSC-SE group. The AD brain is hyperinflammatory due to abnormally deposited plaques, and most neuroimmune cells are activated; however, CNSC-SE treatment resulted in local activation of neuroinflammatory cells only around the plaques. The anti-inflammatory effects of CNSC-SE in the AD brain affected microglial activation Excessive neuroinflammatory reactions in AD promote synaptic loss and cognitive deficiency, which are correlated with active microglial cells confirmed by the morphological changes of Iba-1-positive cells (Fig. 6). This was confirmed by the Western blots of Iba-1 (Fig. 4a, b). However, CNSC-SE treatment reduced both GFAP- and Iba-1-positive cells in the whole brain and in the cortex (Figs. 5c, 6c). While many studies on microglial activation mostly relied on Iba-1 staining to characterize its morphology, quantity, and distribution, it was previously reported that Iba-1 expression (protein and mRNA) might not reflect microglial activation and this still remains a debatable issue [57–59]. Increased Iba-1 in activated microglia has been reported [59, 60], but several studies found that the activation of microglia in brain tissue is not always accompanied by increased expression of Iba-1 [57, 59, 61]. It is suggested that Iba-1 can only identify microglia and its expression may not relate to microglial activation in the brain tissue [62, 63]. Although Iba-1 has been reported as a microglia/macrophage specific marker [64, 65], most studies have relied on immunostaining to determine the extent of microglial activation by checking its morphology and distribution [57–59]. It is still debatable whether increased protein expression of Iba-1 can be used as a sensitive indicator of microglial activation [66]. Therefore, further confirmation with additional markers such as CD11b or ICAM-1 might be necessary and the same might be applicable in the case of astrocytes as well.

Our human cytokine and growth factor arrays showed that IGFBP-2 was present at a higher level in the CNSC-SE than in the iPSC-SE. IGFBP-2 is the most abundant type of IGFBPs in the cerebrospinal fluid, the developing brain and the hippocampus and cortex [43]. Also, IGFBP-2 is a pleiotropic polypeptide that functions as a autocrine and/or paracrine growth factor [43]. Mice with depressive-like behavior caused by chronic immobilization stress showed decreased IGFBP-2 expression in the central amygdala [42]. Also, prenatal stress resulted in reduced IGFBP-2 expression in the hippocampus and frontal cortex in adult male rats [67]. Previous studies also suggest that IGFBP-2 may enhance regenerative sprouting and contribute to neuronal repair in a rat model of sensory spinal axonal injury [68].

IGF-2 is involved in memory enhancement [41]. While both IGF-1 and IGF-2 can be regulated by IGFBP-2, only IGF-2 expression correlates with the expression of IGFBP-2 throughout the CNS development, suggesting that IGFBP-2 may regulate the function of IGF-2 in the CNS [69]. When relatively higher IGFBP-2 concentrations were administered with IGF-2, increases in the percentage of neurite-bearing cells and the average neurite length were confirmed [42]. The ability of IGFBP-2 to improve neuronal survival is related to its role in apoptosis inhibition [43]. The expression of Bcl-2 and IGF/IGFBPs was localized at the same site in the hippocampus, suggesting that the IGF/IGFBPs system and the pro-survival proteins protect the cells from apoptosis and play a critical role during brain development [70]. IGFBP-2 is also significantly increased around the injury site [71].

On the other hand, a possible link between increased IGFBP-2 and mitochondrial dysfunction in AD has been suggested [72]. Blood protein analysis showed increased IGFBP-2 levels in serum before the onset of clinical AD features [73]. Elevated plasma IGFBP-2 levels are suggested to be linked with lower hippocampal volumes [74, 75]. Interestingly, smaller hippocampal volumes are associated with higher IGFBP-2 levels only in the amyloid-negative individuals [74]. IGFBP-2 may differentially modulate normal physiological and pathological functions [76]. IGFBP-2 can be found in extracellular matrix, plasma, and nucleus [77]. Circulating plasma is suggested as a novel biomarker for various brain diseases [75]. Therefore, the detection of plasma IGFBP-2 in AD mice with or without CNSC-SE treatment might be useful for further analysis of this study. Also, the multiple functional domains of IGFBP-2 are thought to contribute to the spatial regulation of IGFBP-2 tumor biology, inducing different regulatory mechanisms operating in the extracellular, intracellular, and nuclear environments [77]. Therefore, further investigations of IGFBP-2 high expression in AD and high presence in the CNSC-SE might be interesting.

Metabolomics represents the final results of interactions between genes, RNAs, and proteins, therefore it has several advantages over other analysis [46, 78]. Comparison of normalized semi-quantitative metabolite profile of CNSC-SE versus neural basal medium using principal component analysis showed a clear distinction, which indicates that CNSC-SE and neural basal medium are biochemically distinct (Fig. 8b). In addition, 47 (36.2%) of the 130 metabolites were present only in CNSC-SE, including products with neuroprotective effects, such as GSH [49, 50] and creatine [51–53]. As such, it can be confirmed that the CNSC-SE contains several components that exert neuroprotective functions or amplify the effect of cognitive function recovery. Furthermore, we report that CNSC-SE contains various components with neuroprotective, anti-amyloid, memory-restoring effects, and excessive neuroinflammatory response-reducing effects.

In conclusion, we found that the iPSC-derived CNSC-SE enhanced neurorestorative activity of cells than MSC-SE with conventional anti-amyloid effects. The efficacy of CNSC-SE was confirmed both in vitro and in vivo, and the memory restoration, which is an important point in the treatment of AD, was significantly enhanced by CNSC-SE. Although these results clearly demonstrate the paracrine action of CNSC-SE, further studies are needed to conclude the process by which CNSC-SE exerts its therapeutic effects on AD. Further investigation of the components of CNSC-SE identified through metabolomics analysis is also warranted.

## Conclusions

In this study, we showed the therapeutic effects of iPSC-derived CNSC-SE in AD. The CNSC-SE delivered via intranasal injection created an anti-inflammatory environment and showed beneficial effects in the complex neuroinflammatory and neurotoxic environment of the AD brain. Possible therapeutic candidates included in CNSC-SE were suggested as potential treatment options. While there are several details that still require further analysis, this study proposes a new therapeutic strategy using human iPSCs for future therapeutic developments for AD. To the best of our knowledge, this is the first study to report the inclusion of multiple neuroprotective and antacid functions in the secretome of iPSC-derived CNSCs, demonstrating that CNSC-SE has high therapeutic potential for AD.

### Supplementary Information

**Additional file 1: Fig. S1.** Differentiation of cortical neurons from hiPSCs. **Fig. S2. **Weight change of 5×FAD mice treated with MSC-SE or CNSC-SE. **Fig. S3. **Intranasal delivery of iPSC-derived CNSC-SE reduces the burden of amyloid beta in the ventral hippocampus of 5×FAD mice.

**Additional file 2**: Recording of Barnes maze test for wild-type mice.

**Additional file 3**: Recording of Barnes maze test for 5×FAD mice.

**Additional file 4**: Recording of Barnes maze test for 5×FAD mice with MSC-SE treatment.

**Additional file 5: **Recording of Barnes maze test for 5×FAD mice with CNSC-SE treatment.

## Data Availability

All data are included in the article and in the supplementary materials.

## Abbreviations

AD: Alzheimer’s disease
Aβ: Amyloid beta
NSC: Neural stem cell
MSC: Mesenchymal stem cell
CNSC: Cortical neural stem cell
iPSC: Induced pluripotent stem cell
CNSC-SE: Cortical neural stem cell secretome
NEUN: Neuronal nuclei
vGlut: Vesicular glutamate transporter
MAP2: Microtubule associated protein 2
BDNF: Brain-derived neurotrophic factor
VEGF: Vascular endothelial growth factor
MEA: Microelectrode array
APP: Amyloid precursor protein
pTau: Phosphorylated tau
BACE: Beta-site APP cleaving enzyme
IL-1β: Interleukin-1β
TNFα: Tumor necrosis factor-α
GFAP: Glial fibrillary acidic protein
iPSC-SE: IPSC secretome
PCA: Principal component analysis
GSH: Glutathione
