# Supplementary material for: Intranasal administration of induced pluripotent stem cell-derived cortical neural stem cell-secretome as a treatment option for Alzheimer’s disease
Source: Transl Neurodegener. 2023 Nov 9;12:50. doi: 10.1186/s40035-023-00384-8 (PMC10634159; doi:10.1186/s40035-023-00384-8)
Supplement: Supplementary file 1 — Additional file 1: Fig. S1. Differentiation of cortical neurons from hiPSCs. Fig. S2. Weight change of 5×FAD mice treated with MSC-SE or CNSC-SE. Fig. S3. Intranasal delivery of iPSC-derived CNSC-SE reduces the burden of amyloid beta in the ventral hippocampus of 5×FAD mice. [file 40035_2023_384_MOESM1_ESM.docx]

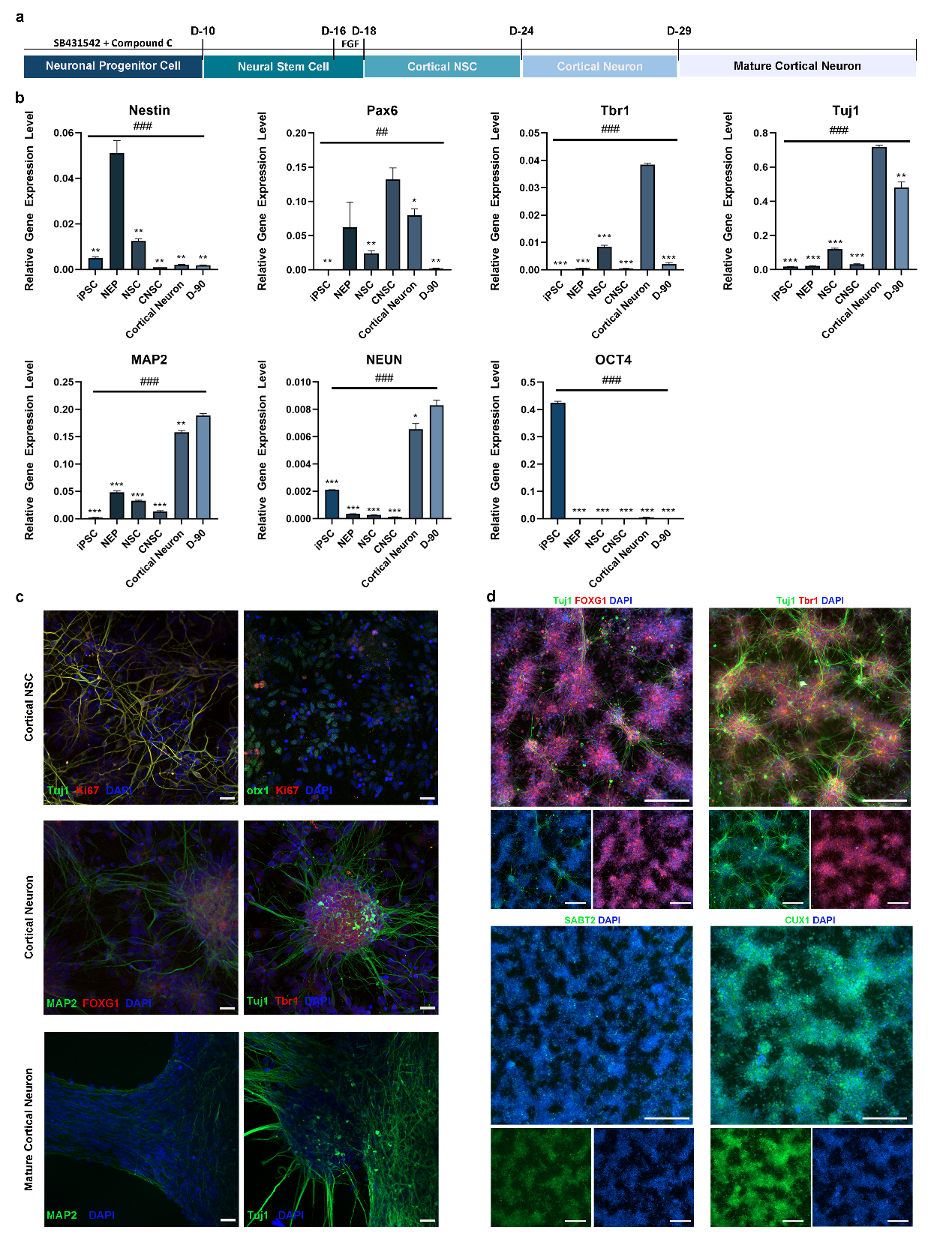


**Fig. S1 Differentiation of cortical neurons from hiPSCs.** (a) Differentiation of cortical neurons derived from human induced pluripotent stem cells (hiPSCs). (b) Gene expression by Realtime PCR (q-PCR), normalized to that of GAPDH: neural progenitor cell marker, Nestin; cortical NSC marker, PAX6; deep-layer neuron marker, TBR1; neuronal markers, MAP2, NeuN, and TUJ1; pluripotency marker, OCT4. Confocal microscopy image to confirm the cortical identity; (c) neuronal proliferation marker Ki67, cortical stem cell marker otx1, and neuronal marker Tuj1 at day 24. Neuronal dendrite marker MAP2, forebrain marker FOXG1, and cortex marker Tbr1 at day 29, (e) Tuj1^+^ and FOXG1^+^ deep layer neurons at day 50 that also express neuronal dendrite marker, MAP2. (d) Cytation5 images to confirm the cortical NSC ; neuronal marker Tuj1, Cortical identity is characterized by co-expression of Pax6, Foxg1, SABT2 and CUX1.

Data are presented as the mean ± SEM. One-way ANOVA; **P* < 0.05; ***P* < 0.01; ****P* < 0.001, Dunnett’s test. Scale bars, 20 μm


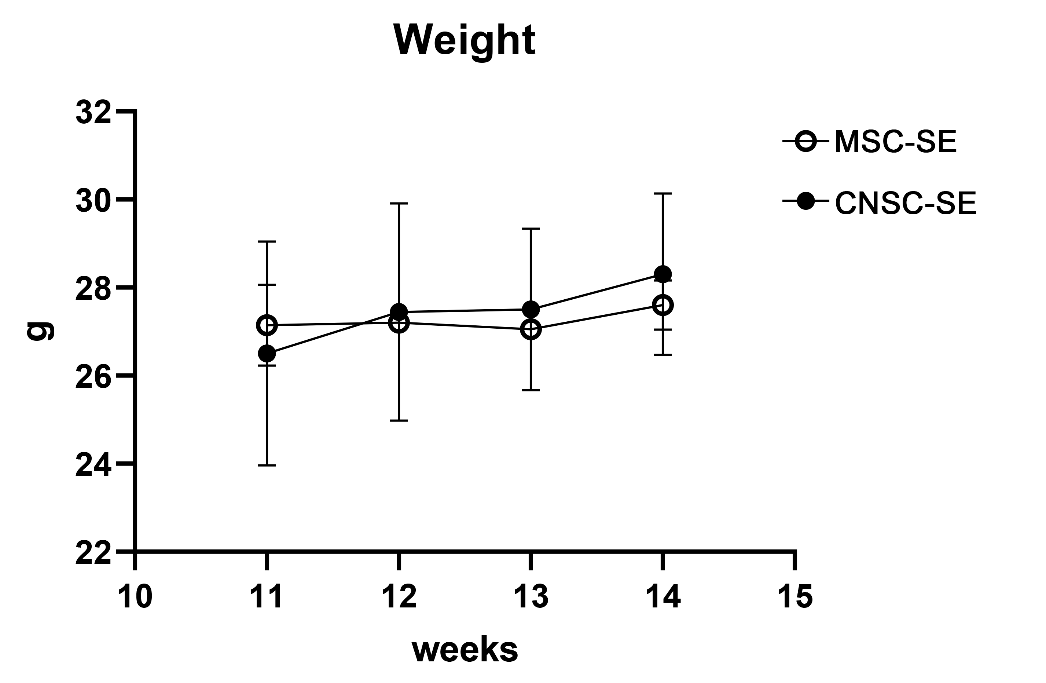


**Fig. S2 Weight change of 5×FAD mice treated with MSC-SE or CNSC-SE.**


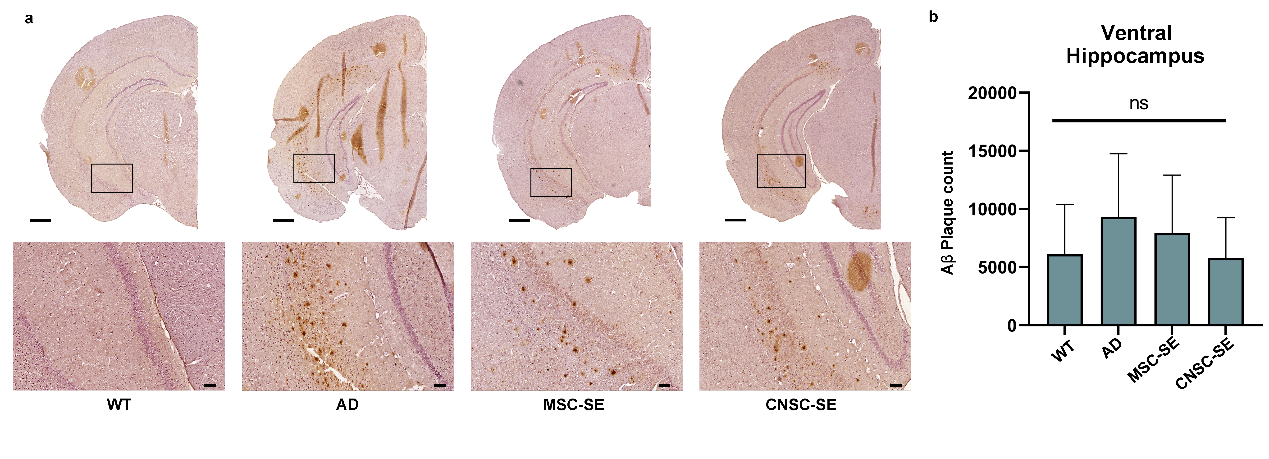


**Fig. S3 Intranasal delivery of iPSC-derived CNSC-SE reduces the burden of amyloid beta in the ventral hippocampus of 5×FAD mice.** (a) Brain sections were stained using immunohistochemistry with Aβ monoclonal antibody in the following groups: (i) Wild type mice as controls (WT group); (ii) AD model of 5×FAD mice (AD group); (iii) CNSC-SE-treated 5×FAD mice; and (iv) MSC-treated 5×FAD mice, (b) Amyloid plaques were counted in the areas count, with clustering in the cortex subiculum by using Image J (measured three times with same place). Scale bars, 200 μm
